# Genetic homogeneity of the critically endangered fan mussel, *Pinna nobilis*, throughout lagoons of the Gulf of Lion (North-Western Mediterranean Sea)

**DOI:** 10.1038/s41598-021-87493-4

**Published:** 2021-04-08

**Authors:** Claire Peyran, Emilie Boissin, Titouan Morage, Elisabet Nebot-Colomer, Guillaume Iwankow, Serge Planes

**Affiliations:** 1 PSL Research University: EPHE - UPVD - CNRS, USR 3278 CRIOBE, 66860 Perpignan, France; 2 Laboratoire d’Excellence «CORAIL», Perpignan, France; 3 Centro Oceanográfico de Baleares, Instituto Español de Oceanografía (IEO), Muelle de Poniente S/N, 07015 Palma de Mallorca, Spain

**Keywords:** Population genetics, Conservation biology

## Abstract

The fan mussel, *Pinna nobilis*, endemic to the Mediterranean Sea, is a critically endangered species facing mass mortality events in almost all of its populations, following the introduction of the parasite *Haplosporidium pinnae*. Such a unique pandemic in a marine organism, which spreads rapidly and with mortality rates reaching up to 100%, could lead to the potential extinction of the species. Only few regions, involving lagoon habitats, remain healthy throughout the entire Mediterranean Sea. This study describes the genetic structure of *P. nobilis* across the Gulf of Lion, including confined locations such as lagoons and ports. A total of 960 samples were collected among 16 sites distributed at 8 localities, and then genotyped using 22 microsatellite markers. Genetic diversity was high in all sites with mean allele numbers ranging between 10 and 14.6 and with observed heterozygosities (*Ho*) between 0.679 and 0.704. No genetic differentiation could be identified (*F*_*ST*_ ranging from 0.0018 to 0.0159) and the percentages of related individuals were low and similar among locations (from 1.6 to 6.5%). Consequently, all fan mussels, over the entire coastline surveyed, including those in the most geographically isolated areas, belong to a large genetically homogeneous population across the Gulf of Lion. Considering the ongoing mass mortality context, this result demonstrates that almost all of the genetic diversity of *P. nobilis* populations is still preserved even in isolated lagoons, which might represent a refuge habitat for the future of the species.

## Introduction

Recent extinction rates have been shown to be 100 to 1000 times their pre-human levels in taxonomically diverse groups through a wide variety of different habitat types, and they continue to accelerate^1^. These extinctions are mainly caused by anthropic activities such as pollution, artificialization, overexploitation, habitat loss and global change, which can lead to the isolation of populations, reduction in their size, and thus a loss of genetic diversity^2^. The conservation genetics concept has been pushed forward in the context of population genetics with the goal of better understanding the dynamics of genes in populations to avoid extinctions. The idea is to apply genetic methods to the conservation and restoration of biodiversity, with genetic diversity being the proxy for the level of extinction risk. Genetic diversity enhances the stability and reliability of ecosystems by providing biological insurance against environmental change^3^. Hence, maintaining a high level of genetic diversity is one of the main targets of conservation biology. Knowledge concerning the level and the distribution of genetic diversity within and among populations of a given species, and on the processes that ensure its maintenance are now more than ever, necessary in order to design conservation strategies.

In marine systems, coastal environments are generally viewed as discontinuous and most organisms are sessile or have very limited movement abilities. There is thus a strong interaction between habitat fragmentation of coastal environments and species life-history traits that can affect dispersal and population connectivity, as found for the pelagic early life stage which represents the main opportunity for movement^4^. This dispersal phase allows connectivity between distant areas and is thus a critical feature of the ecology of marine organisms and evolution, as it drives the genetic structure and composition of populations^5^. The degree of connectivity between populations has an impact on local population survival through replenishment processes, the maintenance of high levels of diversity and by determining their persistence and resilience in case of local disturbance^6,7^. Enhancing dispersal will certainly favor the long-term persistence of species, in a framework where dispersal will be affected by parameters such as habitat fragmentation, effective population size, anthropic activities and climate change^8,9^.

The fan mussel, *Pinna nobilis* (Linnaeus, 1758), is a bivalve endemic to the Mediterranean Sea, that lives half-buried in soft-bottom habitats, generally covered by seagrass meadows. Because it is associated with seagrass meadows, *P. nobilis* is restricted to subtidal areas, up to 30 m deep, as beyond the meadow development is limited by light intensity and water transparency. As a consequence, *P. nobilis* distribution is usually aggregative and fragmented. Even if this association is generally accepted, very dense aggregations of the species were recently reported in lagoons or in artificial and degraded habitats such as ports, which puts into question the species’ habitat requirements^10^.

*P. nobilis* is a successive hermaphrodite organism with asynchronous gamete maturation, thus preventing self-fertilization^11^. Several consecutive spawnings occur during summer, producing millions of veliger larvae. For *P. nobilis*, larval duration has been estimated to be around 5 to 10 days during which larvae are spread by currents^12^. However, there remains a significant lack of knowledge concerning the *P. nobilis* life cycle, as a recent study has shown that the larval stage could last up to 20 days under controlled conditions^13^, a much longer larval duration which would certainly enhance dispersal capabilities.

The species has been overexploited for years, for consumption or for jewelry fabrication. Over the last decades, human activities (anchoring, pollution, habitat reduction and fragmentation) have kept *P. nobilis* populations under high pressure which has led to population decay^14^. Since 1992, the fan mussel has been under strict protection, according to the European Council Directive 92/43/EEC (Annex IV) and the national laws of most Mediterranean countries. In recent years, *P. nobilis* abundance has increased and dense populations have been observed in anthropized habitats, such as lagoons and ports, which were previously thought to be unfavorable to their settlement as the species is generally considered as a bioindicator of the quality of coastal waters^15^. However, despite its endangered status, only few studies have investigated population genetics and connectivity patterns in *P. nobilis*^16–21^. Furthermore, information on the contemporary dynamics is limited, as none of the previous studies integrated the large populations recently described in lagoons.

Today, the species is facing a major ongoing pandemic that threatens its survival. Starting in October 2016, mass mortalities have been observed in fan mussel populations, probably caused by the protozoan parasite, *Haplosporidium pinnae*^22^. However, the causes of the outbreak are far from being completely understood as one recent study supported the occurrence of a multifactorial disease with non-species-specific pathogens that could have triggered mass mortalities in *P. nobilis* populations^23^ whereas another study stated that *H. pinnae* is the only pathological agent considered as essential for the onset of the mortality^24^. The very first signs of mortality were observed in southeast Spain^25^ and the epidemic has now spread throughout the entire Mediterranean Sea^26^. To date, most of the infected populations have been devastated. This is an unprecedented situation for which neither the mortality rates (around 100%) nor the speed of propagation^23,26,27^ have ever been recorded for a marine species, and it could lead to the potential extinction of *P. nobilis*. Following this critical situation, the status of the species has been reevaluated and has been recently moved to “critically endangered” on the IUCN red list^28^. However, a couple of populations in localities along the early infected Spanish coastline remain less affected by the parasite mortality (Alfacs Bay and Mar Menor, Spain^22,26^). Even if the reasons are not yet well understood, greater survival rates might be linked to salinity ranges in those areas compared to the open sea^26^: Alfacs Bay is subjected to high freshwater inflow of the Ebro River whereas Mar Menor is a confined coastal lagoon where salinity can reach up to 40 PSU. Even if observations are limited, lagoons are now a priority habitat in Spanish conservation plans, as large populations have recently been described in this habitat type throughout the Mediterranean Sea.

While accounting for about 250 km of coastline in the Mediterranean Sea, the Gulf of Lion is characterized by numerous lagoons with various hydromorphological characteristics^29^. Recent surveys in these habitats revealed the presence of *P. nobilis* in high densities, even much higher than those observed in the usual seagrass meadows (T. Morage, pers. com.). Recent monitoring data demonstrated that almost 90% of the population of *P. nobilis* is found in the lagoons of Thau and Salses-Leucate along the Gulf of Lion^10^ and, to date, these areas are still free from parasite contamination. Considering the current pandemic situation for *P. nobilis,* these lagoons may represent a key habitat in a conservation strategy and may provide hope for the persistence of the species. In the meantime, as these lagoons are unstable environments, facing variations in nutrient concentrations or fresh water inflows^29^, the long-term survival of the species may be uncertain. Lastly, these lagoons are rendering the distribution of *P. nobilis* highly fragmented and isolated in small patches, and require better knowledge about the population structure and dispersal patterns of the species. Here, we used highly polymorphic markers to (i) assess the genetic diversity of fan mussel aggregations in the different types of habitats, (ii) estimate whether the habitat fragmentation tends to create genetic differentiation and spatial genetic structuring between aggregations and (iii) assess the regional patterns of dispersal throughout the Gulf of Lion. This work is the first genetic study of *P. nobilis* populations in the highly fragmented environment of the Gulf of Lion where lagoon habitats were also considered, before the spread of the pandemic along the French Mediterranean coast.

## Materials and methods

### Sampling

**Figure 1:**
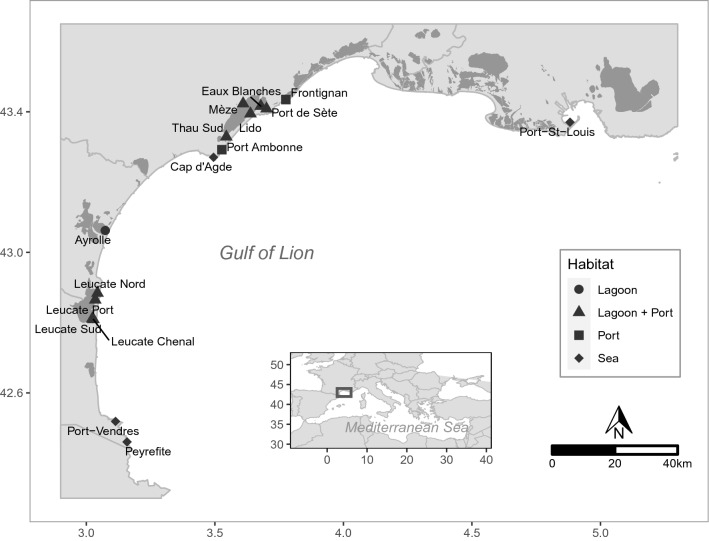
Location of 16 sampled sites for *Pinna nobilis* in the Gulf of Lion, north-western Mediterranean Sea. This map was created using R 4.0.3 (https://www.r-project.org/) using coastline and surface water data provided by https://www.data.gouv.fr/fr/.

As *P. nobilis* is an endangered species, we received necessary permissions for sampling from the DREAL (Direction Régionale de l’Environnement, de l’Aménagement et du Logement) of Occitanie (prefectural order n°2018-s-24). All Methods were carried out in accordance with relevant guidelines and regulations. Samples were collected from all sites where aggregations of *P. nobilis* were observed following a year of extensive and systematic searching in all habitats along the coast of the Gulf of Lion, from the Spanish border to the Rhône estuary. A total of 960 tissue samples were collected from 16 sites along the coast during the summer of 2018 (see Fig. 1 and Table 1 for collection information). We assume that these 16 sites provide a fairly complete sampling of the majority of the main aggregations of *P. nobilis* in the area. Sampled sites were segregated by location, type of habitat (sea, lagoon, port or lagoon linked to the sea via a port) or population density for further genetic analyses. Sites were structured in 8 localities which refer to the geomorphological entities where they were sampled (Table 1). For each site, SCUBA divers biopsied ~ 1 cm^3^ of mantle tissue on random individuals, without moving the individual. This method was shown to be non-lethal after an initial test and survey (T. Morage, pers. com). As a preventive measure, the smallest individuals were not sampled to avoid lethal biopsies. Each tissue sample was stored in absolute ethanol at room temperature.

**Table 1:** Collection information for the 16 sampling sites in the Gulf of Lion (north-western Mediterranean Sea) where the 960 *Pinna nobilis* individuals were collected for this study.

Geomorphological entity	Site	Type of environment	Number of individuals sampled	Population density (/100 m^2^)	Estimated abundance	Percentage of abundance sampled (%)	GPS	
							Longitude	Latitude
Côte des Albères	Peyrefite	Sea	157	1.25	17,000	1	3.158431	42.460506
	Port-Vendres		19	0.15			3.113699	42.518787
Leucate	Leucate Chenal	Port + Lagoon	50	4.58	140,000	0.2	3.02771	42.808951
	Leucate Sud		53	14.5			3.02322	42.81098
	Leucate Port		105	70.83			3.03474	42.86486
	Leucate Nord		54	7.72			3.043567	42.884103
Ayrolle	Ayrolle	Lagoon	51	0.63	2500	2	3.07408	43.06163
Cap d'Agde	Cap d'Agde	Sea	116	4.83	200	58	3.49488	43.27064
Port Ambonne	Port Ambonne	Port	35	1.22	50	70	3.5272	43.29226
Thau	Thau Sud	Port + Lagoon	47	7.29	90,000	0.2	3.544564	43.328782
	Lido		54	1.42			3.638553	43.394586
	Mèze		24	1.59			3.609889	43.423402
	Eaux Blanches		34	2.64			3.678688	43.41625
	Port de Sète		61	4.03			3.701066	43.408866
Frontignan	Frontignan	Port	49	5.97	150	32.7	3.776224	43.434929
Port-St-Louis	Port-St-Louis	Sea	51	1.74	4000	1.3	4.88147	43.37038

### Size estimation

The size of each individual sampled was calculated to identify different size groups that will correspond to fan mussels that settled over successive years and which could therefore represent different clouds of larvae. Because *P. nobilis* lives half-buried in the sediment, it is impossible to directly measure the total length of the shell and thus the size of each sampled individual had to be estimated from three morphometric measurements: the maximum width, the minimum width and the unburied length, as described in Garcia-March et al.^30^. The following equation, described in De Gaulejac^31^, is used to determine the total length:

$$\mathrm{log }(Ht)=\mathrm{0,4003}+\mathrm{0,5028}.\mathrm{log }(H)+\mathrm{0,26}.\mathrm{log }(l)+\mathrm{0,2171}.\mathrm{log }(L)$$

where *Ht* is the total length; *H* the unburied length; *l* the minimum width and *L* the maximum width.

The size estimates were used to further differentiate size groups and to investigate genetic structure in relation to size structure in order to search for genetic heterogeneity of recruitment.

### Molecular analyses

Extraction of genomic DNA from the 960 samples was performed using the QIAcube HT robot following the manufacturer’s instructions (Qiagen, Hilden, Germany). All samples were genotyped using 30 microsatellite markers from Peyran et al.^32^ and González-Wangüemert et al.^33^.

PCRs were performed following the same method as described in Peyran et al.^32^. Microsatellite markers were amplified using Type-it Microsatellite PCR kit (Qiagen, Hilden, Germany) in a final volume of 12.5 µL including 4 µL Type-it Multiplex PCR Master Mix (2X), 6 µL RNase-free water, 1 µL of each primer (2 µM forward and reverse primers diluted in TE pH 8 buffer) and 1.5 µL of DNA template with a concentration of around 50 ng/µL. PCR programs consisted of an initial denaturing step of 5 min at 95 °C, followed by 40 cycles of 30 s at 95 °C, 1 min 30 s at annealing temperature (53–63 °C, depending on the locus^32,33^), 30 s at 72 °C and a final elongation step at 60 °C for 30 min. Loci were combined in multiplex panels according to their allele size and primer annealing temperature. Individuals were genotyped by assessing the allele size. Forward primers were labelled with fluorescent dyes (PET, NED, VIC, 6-FAM, Applied Biosystems) and PCR products were sent to a private company, GenoScreen (Lille, France), for fragment genotyping where they were visualized using an Applied Biosystems 3730 Sequencer. GeneScan 500 LIZ (Applied Biosystems) was used for accurate sizing. Allele sizes were scored and checked manually using GENEMAPPER v.5 (Applied Biosystems). Samples presenting ambiguous peak profiles were re-amplified, genotyped and re-scored and all peak profiles that were still unclear were treated as missing data.

### Data analysis

As mollusks often present amplification troubles during PCR, the quality of the microsatellite markers was investigated using MICROCHECKER v 2.2.3^34^ to search for the presence of null alleles, scoring errors and large allele dropout. On the original panel of 30 markers, eight loci presented evidence of null allele high frequencies and were removed from the study to avoid any bias in further analyses. The final set of markers contained 22 polymorphic loci (Supplementary Table S1).

A preliminary genetic analysis was performed to confirm that some sites within the same area could be agglomerated into localities. As no genetic differentiation was identified (Supplementary Table S2), samples were grouped into 8 localities considered as units for further analyses.

Genetic diversity was investigated using GenAlex 6.503^35^, for each locality and between size groups within locality through mean allele numbers (*Na*), number of private alleles (*Ap*), and expected (*He*) and observed (*Ho*) heterozygosities. As sample size ranged from 35 to 262 across localities, standardized allelic richness (*Ar*) and standardized private allelic richness (*Apr*) were estimated using ADZE software^36^, based on a standardized sample size of 35 (smallest sample size). Hardy–Weinberg exact test was also performed for each loci using GENEPOP software^37^. The p-value of the exact test of Hardy–Weinberg was estimated based on a Markov Chain algorithm (dememorization = 10,000, number of batches = 1000 and iterations per batch = 10,000). Values for inbreeding coefficient (*F*_*IS*_*)* were calculated for all loci and for each localities using the method of Weir and Cockerham^38^, implemented in Genetix ^39^ and significance of values was established by permutations (1000 permutations per population). Differentiation index (*F*_*ST*_*)* was calculated between locality pairs and between size groups by using the Robertson and Hill estimator for *F*_*ST*_^40^ corrected by Raufaste and Bonhomme^41^, RH’, implemented in Genetix, as this estimator is unbiased and shows a lower variance when values of *F*_*ST*_ are low (< 0.05). The Bonferroni sequential correction for multiple tests was then applied to correct the p-values^42^.

An exploration for any genetic structuring was also performed with a MultiDimensional Scaling (MDS), using the *stats* package implemented in R software v. 1.1.453^43^ and based on the matrix of Nei’s genetic distances between localities which was calculated using GenAlex. The molecular genetic variance was analyzed between and within localities using the AMOVA framework in Arlequin suite v. 3.5^44^.

Genetic structure was also explored using a model-based clustering method implemented in STRUCTURE 2.3.4^45^. An admixture model with no prior information about sampling location was used with a burn-in period of 50 000 iterations, followed by a 100,000 Monte Carlo Markov Chain (MCMC) replicates for K = 1 to K = 8 clusters and 10 iterations for each K. The choice of the most likely number of clusters (K) was made by calculating an ad hoc statistic (ΔK) using Structure Harvester online^46^ and based on the rate of change in the log probability of data between successive K as described by Evanno et al.^47^. Several authors pointed out that uneven subpopulations sampling may bias STRUCTURE results and lead to overestimate the number of clusters^48^. StructureSelector^49^, a web based software, was thus also used to estimate the number of clusters (K) based on four estimators, MedMedK, MedMeaK, MaxMedK and MaxMeaK, developed by Puechmaille^48^ and that were shown to be less sensitive to uneven sampling.

A Mantel test was performed to test the isolation by distance hypothesis, based on geographic (distances with straight lines or those which followed the coastline were both tested) and Nei’s genetic distances between locations. The analysis was conducted using the R package *ape*^50^.

Lastly, the genetic relatedness was calculated between all pairs of individuals by the *r*_*xy*_ coefficient^51^ using the R package *Demerelate*^52^ to investigate family structure within localities and within sites. The relatedness coefficient indicates the proportion of shared alleles between two individuals. Thus, with a sufficient number of highly polymorphic loci, a *r*_*xy*_ > 0.25 between two individuals assumes that they have at least one parent in common. The percentage of individuals sharing at least one parent (*Pr*_*xy*_) was calculated within each locality (i.e. the percentage of relations with *r*_*xy*_ > 0.25) and for each size group that could be identified in some localities. This percentage gives a proxy of the admixture in each locality as relatedness indexes are calculated between individuals of different size classes. Because the percentage of individuals biopsied in relation to the estimated abundance is different among localities and very low in some cases (Table 1), the effects of sampling effort on the final results needed to be estimated. We thus modeled *Pr*_*xy*_ depending on the number of individuals in a given sample, using a repeated random sub-sampling method, similar to rarefaction curves. For each locality, new datasets of 10 individuals were created by randomly selecting individuals in the existent database. Other datasets were then created by successively adding individuals that were randomly drawn (without replacement) in the existing database (3 individuals for localities where sample size was small, or 5 individuals for localities where sample size was high). For each locality, we thus had new datasets with 10, 13, 16, etc. or 10, 15, 20, etc. individuals until approaching the sample size. The procedure was repeated five times to increase the number of new datasets. Relatedness coefficients were calculated between all pairs of individuals within each of these new datasets and *Pr*_*xy*_ was estimated. A non-linear regression was then performed to model the relation between the number of individuals sampled and *Pr*_*xy*_ in each locality in order to estimate the threshold of sampling effort where adding more individuals does not increase the percentage of related individuals found. The non-linear regression was also used to extrapolate the maximum *Pr*_*xy*_ in localities where sample size was too small.

### Ethical approval

The sampling was non-lethal and approved by the DREAL (Direction Régionale de l’Environnement, de l’Aménagement et du Logement) of Occitanie (prefectural order n°2018-s-24).

## Results

### Genetic diversity

13 out of 22 markers (see Table S1) as well as 5 out of 8 localities (Table 1) showed low but significant *F*_*IS*_ values, indicating a significant heterozygote deficiency and departure from Hardy–Weinberg equilibrium. The analyses revealed a high level of genetic diversity as the mean allele number per locality (*Na*) ranged from 10 to 14.6 (Table 2). Standardized allelic richness (*Ar*) was very similar between localities as it ranged from 8.1 to 8.5. The number of private alleles (*Ap*) was higher in Leucate and Thau, which are the two most isolated localities. However, the standardized private allelic richness (*Apr*) ranged from 0.26 to 0.37 and is thus very similar within localities. The observed (*Ho*) and expected (*He*) heterozygosities ranged from 0.679 to 0.704 and from 0.705 to 0.726, respectively. Overall, the number of alleles and the heterozygosity values were very homogeneous among localities and there was no decrease in diversity in isolated lagoon areas.

**Table 2:** Summary statistics of genetic diversity indices of *Pinna nobilis* in each locality.

Locality	*N*	*Na*	*Ap*	*Ar*	*Apr*	*Ho*	*He*	*F*_*IS*_
Côte des Albères	176	13.5	5	8.5	0.37	0.679	0.707	0.02903***
Leucate	262	14.6	20	8.3	0.26	0.685	0.707	0.04461***
Ayrolle	51	10.9	1	8.5	0.28	0.684	0.705	0.05279***
Cap d'Agde	116	12.6	6	8.5	0.35	0.688	0.711	0.01862*
Port Ambonne	35	10.0	1	8.4	0.29	0.691	0.705	0.009
Thau	220	14.3	14	8.3	0.27	0.704	0.726	0.03524***
Frontignan	49	10.5	1	8.1	0.26	0.701	0.707	–0.009
Port-St-Louis	51	10.9	3	8.2	0.29	0.703	0.717	0.007

Significant values of *F*_*IS*_ are indicated with *p-value < 0.05; **p-value < 0.01; ***p-value < 0.001.

*N* number of individuals sampled, *Na* mean number of alleles, *Ap* number of private alleles, *Ar* standardized allelic richness, *Apr* standardized private allele richness, *Ho* observed heterozygosity, *He* expected heterozygosity and *F*_*IS*_ inbreeding coefficient.

### Genetic differentiation

**Figure 2:**
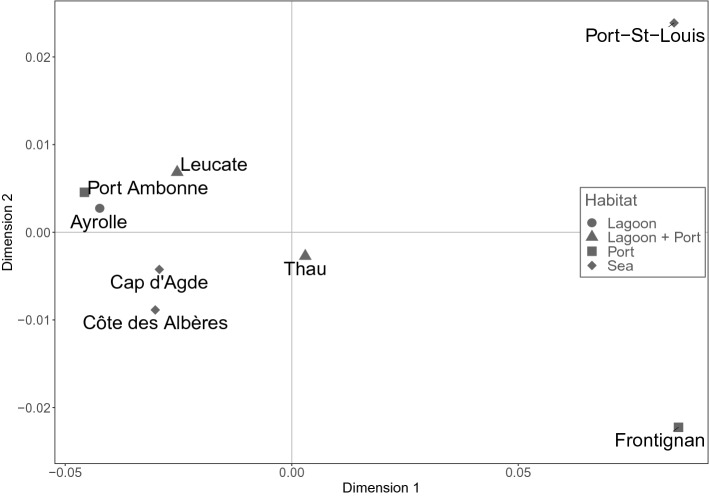
Multidimensional Scaling (MDS) based on Nei’s genetic distances among the 8 localities where *Pinna nobilis* individuals were collected.

All of the pairwise *F*_*ST*_ values were low (ranging from 0.0018 to 0.0159) and only two were significant after Bonferroni sequential correction: between Thau and Leucate and between Thau and Cap d’Agde (Table 3). The multivariate analysis (MDS) based on Nei’s genetic distance (Fig. 2) did not reveal any clear partitioning of the localities. All localities were grouped together with the exception of Port-St-Louis and Frontignan which were distant from all other localities, a result inconsistent with the *F*_*ST*_ values described above. However, as the MDS is a graphical representation, these distances between Port-St-Louis, Frontignan and other localities may not be significant. The AMOVA analyses showed that 99% of the total variation was supported by the variability among individuals (Supplementary Table S3a,b). The model-based clustering method analysis for population structure identified 4 clusters using Evanno’s method and 2 clusters using Puechmaille’s method (Supplementary Fig. S1). However, the analysis did not clearly assign each individual to a distinct cluster and there was a considerable level of admixture that did not allow clusters to be identified in relation to spatial distribution of individuals in localities. The isolation-by-distance test did not reveal any significant correlation between the genetic and geographic distances when distances were calculated using straight lines (Mantel test; Z = 45,468.91; p-value = 0.3863) but was significant with distances following the coastline (Mantel test; Z = 64,534.25; p-values = 0.0209). Overall, none of the analyses revealed a clear and consistent genetic differentiation between sampled localities that can be linked to the geographic localization or habitat type.

**Table 3:** *F*_*ST*_ values of pairwise comparisons (Robertson and Hill estimator for *F*_*ST*_, 1984 corrected by Raufaste & Bonhomme, 2000) between the 8 localities where *Pinna nobilis* specimens were collected.

*F*_*ST*_	Leucate	Ayrolle	Cap d'Agde	Port Ambonne	Thau	Frontignan	Port St Louis
Côte des Albères	0.00181	0.00919	0.00338	0.00634	0.00225	0.0085	0.00773
Leucate		0.00443	0.00407	0.01594	0.01269*	0.00853	0.0099
Ayrolle			0.00397	0.00495	0.00624	0.00771	0.00308
Cap d'Agde				0.01354	0.01952*	0.00866	0.00577
Port Ambonne					0.01181	0.00551	0.00383
Thau						0.01332	0.00846
Frontignan							0.00463

*Indicates significant values after Bonferroni sequential correction.

### Relatedness

**Figure 3:**
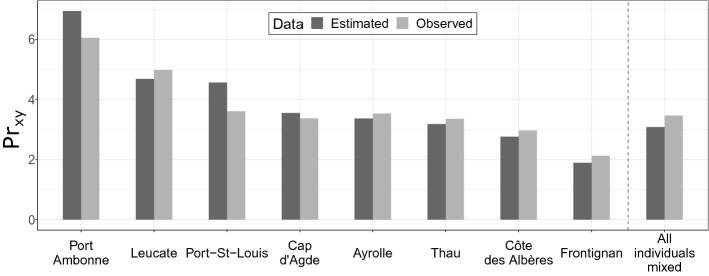
Percentage (*Pr*_*xy*_) of *Pinna nobilis* individuals sharing at least one common parent (i.e. percentage of relations with *r*_*xy*_ > 0.25) calculated for each locality and for all sampled individuals mixed. Based on Queller and Goodnight (1989) *r*_*xy*_ index for relatedness.

The non-linear regression between *Pr*_*xy*_ and sampling effort (Supplementary Fig. S2) showed that all the curves quickly reached an asymptote with a sampling effort of approximately 50 individuals per sample, which is about the minimum sample size we have when considering localities, except in Port Ambonne (35 individuals). *Pr*_*xy*_ was then estimated following the nonlinear regressions obtained for each locality and for all individuals mixed (Fig. 3). *Pr*_*xy*_ was similar among localities (ranging from 2.8 to 4.7%) except for Frontignan (1.6%) and Port Ambonne (6.5%). Similar results were found when calculating *Pr*_*xy*_ within sites (Supplementary Table S4). Then, when considering all individuals within a single sample *Pr*_*xy*_ was similar to the mean percentage in each locality (3.1%).

### Size classes

**Figure 4:**
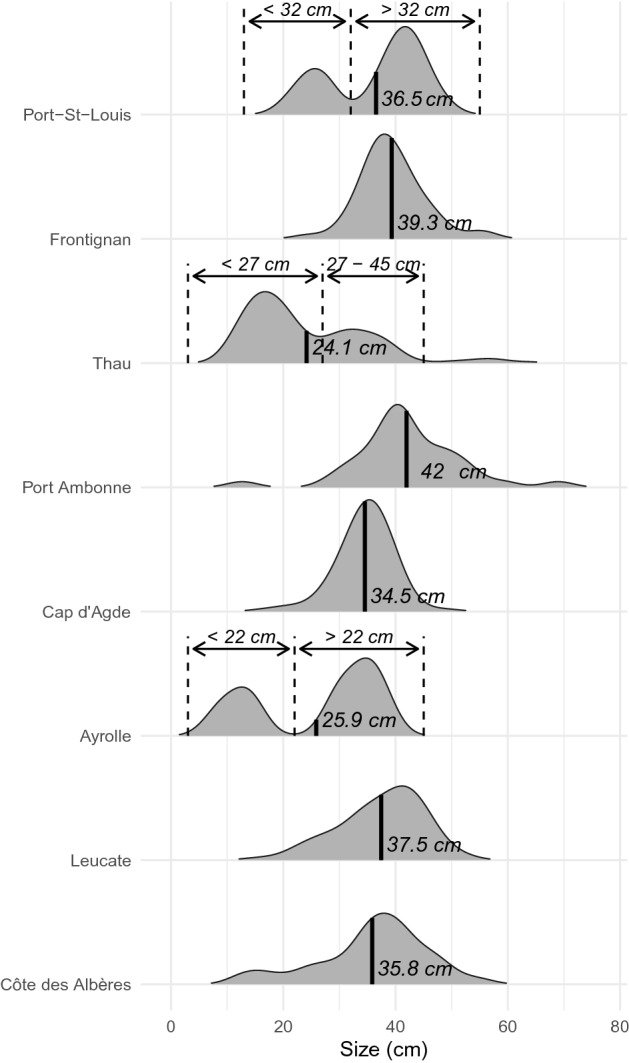
Ridgeline plot showing the size density distribution of *Pinna nobilis* individuals sampled in each locality. Mean sizes in each locality are represented by solid lines. Arrows represent the range of the size groups identified in Port-St-Louis, Thau and Ayrolle.

The mean sizes ranged between 30 and 45 cm except in Thau and Ayrolle where there was a larger number of small individuals (Fig. 4). Two clear size classes were segregated in Thau, Port-St-Louis and Ayrolle (Fig. 4). In the three localities, the genetic diversity was similar between size groups (Table 4) and there was no genetic difference between the two size classes in each locality as all *F*_*ST*_ values were non-significant (Table 4). Then, *Pr*_*xy*_ in each size class were not different from each other and were also similar to *Pr*_*xy*_ values when size classes were grouped as a single sample (Table 4). Only in Port-St-Louis, smaller individuals showed a very low *Pr*_*xy*_ (< 1%).

**Table 4:** Summary statistics for the two size classes in Ayrolle, Thau and Port-St-Louis.

Size groups	*N*	*Na*	*Ho*	*He*	*F*_*IS*_	*F*_*ST*_	*Pr*_*xy*_ (%)
**Ayrolle**							**3.51**
0–22 cm	18	8.9	0.684	0.694	0.04265*	0.01601	4.57
22–50 cm	32	9.2	0.677	0.697	0.06160***		3.02
**Thau**							**3.24**
0–27 cm	114	12.8	0.694	0.713	0.03117***	0.00368	2.73
27–45 cm	55	10.8	0.701	0.710	0.02229*		3.30
**Port-St-Louis**							**3.60**
0–32 cm	16	7.9	0.699	0.683	0.00941	0.01703	0.83
32–50 cm	35	9.8	0.701	0.689	0.00620		4.03

Significant values of F_IS_ are indicated with *p-value < 0.05; **p-value < 0.01; ***p-value < 0.001.

*N* the number of individuals in the size classes, *Na* the mean number of alleles, *Ho* the observed heterozygosity, *He* the expected heterozygosity, *F*_*IS*_ the inbreeding coefficient, *F*_*ST*_ values of pairwise comparisons between size classes and *Pr*_*xy*_ the percentage of individuals sharing at least one common parent (i.e. percentage of relations with *r*_*xy*_ > 0.25) within the two size groups and considering the two size groups mixed (in bold).

## Discussion

The genetic structure of *P. nobilis* across the Gulf of Lion, based on the analysis of 960 fan mussels sampled at 8 localities, with different environmental conditions, appeared homogeneous throughout the area including samples isolated in enclosed lagoons and harbors. High genetic diversity was highlighted in all locations sampled, no significant genetic differentiation was identified and the percentages of related individuals were very limited and similar between all locations. In a given site, genetic parameters were also very homogeneous between size classes. These results suggest that all fan mussels belong to a large, homogeneous and highly connected population at the scale of the Gulf of Lion, which has direct implications for genetics-based conservation strategies hereinafter discussed.

Several loci showed evidence of heterozygote deficiency, which is a common phenomenon in mollusks^53,54^. It could be caused by different factors such as null alleles, inbreeding, Wahlund effect or selection. The possible reasons for heterozygote deficiency in *P. nobilis* were further detailed in a previous study^32^. All the loci that showed evidence of null alleles were removed from the dataset to avoid bias in the results.

### Genetic diversity and relatedness

Genetic diversity was similar among the different localities, except in Thau, Leucate and for the Côte des Albères which showed higher values because the number of private alleles was higher. Such higher genetic diversity likely reflects the larger sample sizes from these three localities (i.e. increasing rare private alleles in these localities) as allelic richness was similar among localities when standardizing sampling effort. The levels of diversity and heterozygosity of *P. nobilis* are similar to those found in other bivalve species: *Cerastoderma glaucum*^55^, *Ostrea edulis*^56^ and *Crepidula fornicata*^57^. Genetic diversity values found here were slightly higher compared to what was found in other *P. nobilis* populations in the Baleares^19^ or along the Spanish coast^20^ but this is also likely due to a lower sampling effort in their studies. This high level of diversity in all sampled localities supports the hypothesis of a large effective population size that maintains high polymorphism, even in the presence of fragmented and isolated habitats^58^. Such hypothesis of large effective population is quite common in marine sessile benthic species that have external reproduction as they synchronize the release of their gametes to ensure a higher probability of external fecundation. This is the case for several bivalves such as the scallop *Pecten fumatus,* where synchronized spawning events are triggered by high density and abundance of conspecifics^59^ and thus annual recruitments involve a large number of spawners. This phenomenon was also observed in other bivalves such as the date mussel^60^ and in other sessile organisms such as sea urchins^61^, brittle stars^62^ and other invertebrates^63^. In the case of *P. nobilis*, the reproduction patterns are quite complex and still poorly understood. For example, Trigos et al.^13^ reported the occurrence of internal fertilization with females that maintain eggs in their body cavity whereas external fertilization was largely admitted as the main mode of reproduction^64,65^. Then, while fan mussels are described as a successive hermaphrodite, 38% of individuals in controlled conditions and 20% in wild population in Alfacs Bay, Spain, appeared simultaneously male and female, leading to self-fertilization^13,66^. Such high rates of self-fertilization would decrease genetic admixture and thus genetic diversity, and could induce higher rates of inbreeding, particularly in enclosed areas such as Thau or Leucate. However, Prado et al.^66^ add caution to their findings by saying that unfavorable ecological situations may have the potential to trigger the development of simultaneous hermaphrodites in bivalves populations. This phenomenon has not been observed in the populations of the Cabrera National Park Marine Protected Area in the Balearic Islands^67^ which also shows evidence of large admixture^19^. Considering the high level of genetic diversity and low inbreeding coefficients that were observed in all localities in the present study, such high rates of self-fertilization are unlikely to occur here and the hypothesis of synchronized spawning events involving a large number of individuals is more relevant with the genetic data gathered thus far.

The percentage of related individuals was not larger in a given locality than considering all individuals mixed in a single sample, even for the most isolated localities such as lagoons. The two extreme values (Frontignan: 1.6% and Port Ambonne: 6.5%) were found in the smallest harbors with the smallest abundances. Low abundances increase the effects of the heterogeneity of larval supply, during recruitment, in both directions, thus leading randomly to more or less related individuals. Overall, only a small proportion of individuals showed relatedness values > 0.25 (mean of 3.3%) emphasizing evidence of large effective population size and substantial admixture during reproductive events. This result also correlates with similarity of the diversity indices among localities. Moreover, in the three sites where two size classes could be identified, individuals did not show more relatedness within a given size group. Only few studies investigated relatedness in wild marine species and to date, none of them have studied relatedness in the Gulf of Lion and within lagoons. However, Costantini et al.^68^ studied the relatedness of red coral individuals and found that 25.98% of pairwise comparisons involved related individuals which is much higher than what we are reporting for *P. nobilis*.

Both diversity indices and relatedness estimates demonstrate that even if patchy and isolated, aggregations of *P. nobilis* originate from large effective population sizes. This is mainly linked to the reproductive mechanism that consists of the synchronic release of gametes by all individuals to ensure success in the fecundation and an increase in admixture. In this framework, the question of connectivity between aggregations remains, since the large population size in lagoons could maintain high genetic diversity indexes while, at the same time, this high diversity in small isolated harbors could indicate a supply from outside populations.

### Regional patterns of dispersal

None of the analyses performed in this study showed genetic differentiation among localities when considering genetic diversity levels, allele frequencies or relatedness index (i.e. combinations of alleles). All fan mussel aggregations belong to a large homogeneous, highly connected, population across the entire Gulf of Lion. Even small aggregations located in isolated areas (lagoons or ports) were not genetically segregated. The absence of spatial genetic differentiation is often interpreted as a signal for significant connectivity but this is primarily an equilibrium between genetic drift and migration. In the context of large effective population size, the genetic drift will be limited and only a small amount of migration is required to maintain genetic homogeneity. Considering the isolated aggregations of *P. nobilis* located in lagoons, the potential for a high self-recruitment rate remains likely as these lagoons have limited water inflow with the open sea which could limit the exportation of pelagic larvae. Then, as we are dealing with a species characterized by large effective population size, in relation to its reproductive mode, only a small amount of larval supply from other localities should be necessary to maintain low *F*_*ST*_ values, limited relatedness and the overall genetic homogeneity. In the meantime, due to their properties, larvae from all localities, even enclosed lagoons, have the potential to be exported to the open sea and spread to all localities, drifted by currents, which would also maintain the overall genetic homogeneity. Both scenarios would explain the low proportion of related individuals, as recruitment is the result of a large reproductive event, involving a large number of spawners, and relatives will not necessarily remain together until settlement (i.e. no kin aggregation).

The absence of genetic differentiation is common in mollusks, particularly at this spatial scale, as species are known to have large effective population sizes and long larval durations (for example in *Concholepas concholepas*^69^, *Crepidula fornicata*^57^ and *Pecten maximus*^70^). Population genetics studies were performed for *P. nobilis* in Balearic Islands and along the Spanish coast and authors reported no, or very weak, genetic differentiation between sites^19,20^. Populations were situated in open sea locations and authors associated the lack of genetic structure to a high connectivity in their study area. Simulations performed by Wesselmann et al.^20^, highlight the high dispersal potential of fan mussels during the pelagic larval state. In the present study, considering the size of the study area and the dispersal potential of larvae, the hypothesis of high connectivity could thus be consistent, at least between the most exposed locations, and even between the most distant ones such as Côte des Albères and Port-St-Louis.

Sea-lagoon genetic connectivity is poorly documented in the Gulf of Lion. To our knowledge, fine-scale genetic structure among lagoons in this area was investigated, in bivalve species, only for *Ruditapes decussatus*^71^ and *Mytilus galloprovincialis*^72^ and, similarly to the present study for *P. nobilis*, no genetic differentiation could be identified. Similar results were also found for the sand goby *Pomatoschistus minutus*^73^. These species are known to have large population sizes. We could thus be in the same situation as that of *P. nobilis*, where it is difficult to differentiate both scenarios of high connectivity* vs* reproductive events involving large number of spawners and low migration which maintains the genetic homogeneity.

Other studies on *P. nobilis* showed isolated populations in Greece^16^ and in the Venetian lagoon^18^, where geographic configuration and environmental conditions are similar to those in the lagoons in the Gulf of Lion. Genetic differentiations can thus occur in isolated areas, when a population is segregated by geography or by marine currents, which create natural barriers and prevent populations from exchanging larvae^16^. Given the particular geographic configuration of the Gulf of Lion’s coast, compartmentalization of populations could be expected, especially for lagoons. Finally, Pérez-Ruzafa et al.^74^ reported that connectivity between the sea and lagoon must be much lower than expected, by simulating larval dispersal of several species with different life history traits. This could thus be a clue to support the hypothesis of high self-recruitment rates in lagoons.

### Implication for conservation

Results from the present study on *P. nobilis* demonstrate genetic homogeneity across localities, wherever they are (lagoons, harbors, open sea) that implies some connectivity among these localities that maintain genetic diversity. The question concerning the contribution of this connectivity within the context of repopulating localities remains, as we could oppose two scenarios involving a dominance of migration *vs* one with a negligible migration and dominance of self-recruitment from large local populations. In the case of large lagoons such as Thau and Leucate, it is likely that self-recruitment dominates the local recruitment since all size classes and more regular settlement patterns are present. Conversely, in the case of small harbors, repopulation is highly dependent on migration that is typically more variable, leading to more variability in size classes.

*P. nobilis* is a sedentary endemic species with a highly fragmented distribution. This study highlights a large genetically homogenous population with high genetic diversity across the entire coast of the Gulf of Lion. This high genetic diversity may involve a large number of spawners and thus the population may have a large effective size. Maintaining a high level of genetic diversity in populations is one of the fundamentals of genetic conservation as it preserve the species’ abilities to adapt to environmental changes and increase resilience^75^. Considering the results of the present study and regarding the pandemic occurring throughout the Mediterranean Sea, preserving fan mussels in a single locality, for example, might allow for the entire genetic variability of fan mussels in the Gulf of Lion to be preserved. Then, as migration between localities, even small, might exist, it could help to repopulate other areas. In a context of developing a species rescue plan, conserving a relatively small number of individuals in aquariums, under controlled conditions to protect them from being infested by the parasite, might be enough to provide a backup of almost the entire genetic diversity of the species.

This study also showed the wide diversity of habitat types that the fan mussels can colonize. The epidemic, caused by the parasite *H. pinnae* and leading to mass mortality, has now spread throughout almost the entire Mediterranean Sea and has already reached the coast of the Gulf of Lion where all of the infected populations have been devastated. To date, only individuals settled in lagoons remain less or unaffected and the survival of the species is likely to rest on them, as there is no longer dense population in the open sea. The present study thus has important implications for conservation as it highlights the opportunity to find a habitat that is suitable for fan mussels but which is unfavorable to the development of the parasite. Lagoons, thanks to their particular physico-chemicals conditions, might thus represent a refuge habitat for fan mussels, which could be the key for the persistence of the species in the wild.

## Supplementary Information

Supplementary Information.
