# Supplementary material for: Genetic homogeneity of the critically endangered fan mussel, Pinna nobilis, throughout lagoons of the Gulf of Lion (North-Western Mediterranean Sea)
Source: Sci Rep. 2021 Apr 8;11:7805. doi: 10.1038/s41598-021-87493-4 (PMC8032772; doi:10.1038/s41598-021-87493-4)
Supplement: Supplementary file 1 — Supplementary Information. [file 41598_2021_87493_MOESM1_ESM.docx]

**Supplementary Materials.**

**Genetic homogeneity of the critically endangered fan mussel, *Pinna nobilis*, throughout lagoons of the Gulf of Lion (North-Western Mediterranean Sea).**

Authors: Claire Peyran^1^, Emilie Boissin^1,2^, Titouan Morage^1^, Elisabet Nebot-Colomer^1,3^, Guillaume Iwankow^1^, Serge Planes^1,2^

^1^ PSL Research University: EPHE - UPVD - CNRS, USR 3278 CRIOBE, 66860 Perpignan, France

^2^ Laboratoire d'Excellence « CORAIL »,

^3^ Instituto Español de Oceanografía (IEO). Centro Oceanográfico de Baleares. Muelle de Poniente s/n, 07015. Palma de Mallorca, Spain

Corresponding author: claire.peyran@gmail.com

**Supplementary Table S1**: Characterization of the 22 microsatellite loci used for this study. T_A_: primer annealing temperature; N: number of individuals with reliable amplification; N_a_: number of alleles; Ho: observed heterozygosity; He: expected heterozygosity; F_IS_: inbreeding coefficient.

| **Locus** | **Primer Sequences** | **Repeat**  **Type** | ***T_A_* (°C)** | **Size range (bp)** | ***N*** | ***Na*** | ***Ho*** | ***He*** | ***F_IS_*** |
| --- | --- | --- | --- | --- | --- | --- | --- | --- | --- |
| **11847** | F : ACACTCCAGTACAAGTCACAAATG | (TGT)_18_ | 53°C | 66 - 177 | 938 | 34 | 0.8774 | 0.8795 | 0.00295 |
|  | R : AGAATGCCTGAGTGGGACAG |  |  |  |  |  |  |  |  |
| **3.2** | F : CCGAGGTCCCGTATCACAG | (ACC)_12_ | 53°C | 176 - 245 | 928 | 19 | 0.8330 | 0.8536 | 0.02465* |
|  | R : TGCCCTTTGTGTCATTATTTCG |  |  |  |  |  |  |  |  |
| **6980** | F : AGTCTGCATAACTTCCACTGC | (ACC)_10_ | 53°C | 215 - 260 | 922 | 16 | 0.7950 | 0.8292 | 0.04176*** |
|  | R : GGCCACCAGTAAGCTCTTTG |  |  |  |  |  |  |  |  |
| **1490** | F : CTTCTGATTGACCGTAAGTG | (AAC)_11_ | 53°C | 173 - 236 | 926 | 19 | 0.7646 | 0.7729 | 0.01135 |
|  | R : AAACTCAGACAACGAGACAA |  |  |  |  |  |  |  |  |
| **15415** | F : ACCACTACTCATGCCTCACC | (AC)_16_ | 53°C | 223 - 271 | 891 | 19 | 0.8496 | 0.8783 | 0.03320** |
|  | R : AACGAAGGTCGGCTATAACG |  |  |  |  |  |  |  |  |
| **12107** | F : TGCATCTGCATCGTGAGG | (CAT)_8_ | 55°C | 104 - 128 | 945 | 8 | 0.2360 | 0.2386 | 0.01165 |
|  | R : TGGTGGTCAAGTGGGATGAC |  |  |  |  |  |  |  |  |
| **15584** | F : TGAGGTGGACGTCGCTTAAC | (TGT)_13_ | 55°C | 223 - 277 | 950 | 19 | 0.8737 | 0.8733 | 0.00007 |
|  | R : GTCGAAGTCTCGTGCGAAAG |  |  |  |  |  |  |  |  |
| **9902** | F : TCTAAGGTGCTTGATGTGGA | (GTCT)_12_ | 55°C | 204 - 256 | 950 | 14 | 0.6411 | 0.6815 | 0.05993** |
|  | R : TTAGACGGAAACCATGTGTG |  |  |  |  |  |  |  |  |
| **4482** | F : TATCCAGCAAGAGCACCAGC | (CAA)_9_ | 55°C | 183 - 210 | 956 | 10 | 0.3609 | 0.3686 | 0.02156 |
|  | R : TGACTTGGGTACTGGGTTCG |  |  |  |  |  |  |  |  |
| **15393** | F : TTGAGTGGGACGTTAAACAA | (CAA)_12_ | 57°C | 171 - 225 | 880 | 16 | 0.6636 | 0.7066 | 0.06140*** |
|  | R : ACGCTCCTGTTTTGTCTTCT |  |  |  |  |  |  |  |  |
| **15096** | F : AGTGCGTTGTTTCAATTTCA | (GT)_12_ | 57°C | 166 - 204 | 949 | 19 | 0.8757 | 0.8825 | 0.00832 |
|  | R : TAGGCAAATTTTCTCGCTCT |  |  |  |  |  |  |  |  |
| **15006** | F : AACAACAGCAACTGCACATC | (CAA)_12_ | 57°C | 195 - 210 | 941 | 5 | 0.2933 | 0.2656 | -0.10383 |
|  | R : ATGCTTGGCACTAATGGTCT |  |  |  |  |  |  |  |  |
| **14331** | F : ATCGGAAATCGGATAGGCTG | (GT)_16_ | 57°C | 76 - 130 | 932 | 25 | 0.8637 | 0.8903 | 0.02950** |
|  | R : TAATGTGAGTTGTGTGGCGG |  |  |  |  |  |  |  |  |
| **8995** | F : AGTGGAAGAGAAGCCATACG | (GTCT)_14_ | 57°C | 226 - 290 | 955 | 17 | 0.7958 | 0.7993 | 0.0049 |
|  | R : TACTTGACGGAAACCATGTG |  |  |  |  |  |  |  |  |
| **5017** | F : ACCGAAGTCGAGATGGTTGG | (GTT)_10_ | 60°C | 201 - 249 | 949 | 16 | 0.7640 | 0.8287 | 0.07863*** |
|  | R : CTTCACTGGCACACGATGC |  |  |  |  |  |  |  |  |
| **4664** | F : ACATGCATACATACAAACAAGTGC | (ACAT)_9_ | 60°C | 127 - 203 | 924 | 18 | 0.8214 | 0.8715 | 0.05800*** |
|  | R : CCTTTCTGCCCGACCTCTC |  |  |  |  |  |  |  |  |
| **5.2** | F : TTGCATGTGCCACCATAATC | (ATAGT)_11_ | 60°C | 169 - 224 | 944 | 12 | 0.5699 | 0.6080 | 0.06312*** |
|  | R : TTCATACCGATGAGCCAAATG |  |  |  |  |  |  |  |  |
| **4.3** | F : TGGATCTAGACTCTTTGTTTGTCTTC | (ATTT)_17_ | 60°C | 238 - 350 | 942 | 29 | 0.8450 | 0.9363 | 0.09807*** |
|  | R : ACAGTGCCATGCTATGTTGC |  |  |  |  |  |  |  |  |
| **14763** | F : AGCATCTGGTAACACGACGG | (TTG)_14_ | 63°C | 134 - 200 | 941 | 22 | 0.6684 | 0.6934 | 0.03652* |
|  | R : TGGCGGCTCTAGAAAGATTG |  |  |  |  |  |  |  |  |
| **3.5** | F : CCTAGCCTACATTCCATATGTGC | (AAT)_13_ | 63°C | 143 - 191 | 876 | 16 | 0.4144 | 0.4281 | 0.03254* |
|  | R : TCATGTCTATGTCAAATGAACTCG |  |  |  |  |  |  |  |  |
| **2287F1R1** | F : AGGTCGAATAGCAATAACAACAA | (ACA)_12_ | 63°C | 89 - 131 | 942 | 15 | 0.8227 | 0.8128 | -0.01223 |
|  | R : GGCGTGGAGGTAAGAGTCAA |  |  |  |  |  |  |  |  |
| **9918R2** | F : GTCTTTCTGTGCCGATGTCTG | (ATCT)_7_ | 63°C | 206 - 254 | 923 | 14 | 0.5645 | 0.7434 | 0.06212*** |
|  | R2 : GGACGGGCCGACCTATCT |  |  |  |  |  |  |  |  |

Significant values of F_IS_ are indicated with *p-value < 0.05; **p-value < 0.01; ***p-value < 0.001.

**Supplementary Table S2**: F_ST_ values of pairwise comparisons between sampled sites (Robertson and Hill estimator for FST, 1984 corrected by Raufaste & Bonhomme, 2000).

| ***F_ST_*** | **Frontignan** | **Leucate Chenal** | **Leucate Nord** | **Leucate Port** | **Leucate Sud** | **Peyrefite** | **Pinnasouk** | **Port de Sète** | **Port-Vendres** | **Port Ambonne** | **Port-St-Louis** | **Thau Eaux blanches** | **Thau Lido** | **Thau Mèze** | **Thau Sud** |
| --- | --- | --- | --- | --- | --- | --- | --- | --- | --- | --- | --- | --- | --- | --- | --- |
| **Ayrolle** | 0.009 | 0.00312 | 0.00454 | 0.00336 | 0.00391 | 0.00512 | 0.00364 | 0.00453 | 0.00688 | 0.00504 | 0.00231 | 0.00496 | 0.0072 | 0.00519 | 0.00585 |
| **Frontignan** |  | 0.01119 | 0.0105 | 0.01458 | 0.00953 | 0.00942 | 0.00908 | 0.00927 | 0.01224 | 0.00485 | 0.00459 | 0.00689 | 0.00529 | 0.01348 | 0.00568 |
| **Leucate Chenal** |  |  | 0.00173 | 0.00202 | 0.00784 | 0.01476 | 0.0047 | 0.01275 | 0.0247 | 0.01174 | 0.00904 | 0.0056 | 0.00529 | 0.00968 | 0.00649 |
| **Leucate Nord** |  |  |  | 0.00318 | 0.00459 | 0.01329 | 0.00529 | 0.00898 | 0.01997 | 0.00491 | 0.00825 | 0.00589 | 0.0041 | 0.00594 | 0.00389 |
| **Leucate Port** |  |  |  |  | 0.00412 | **0.02512*** | 0.00487 | **0.01591*** | 0.01938 | 0.00724 | 0.00828 | 0.00731 | 0.0037 | 0.01042 | 0.00483 |
| **Leucate Sud** |  |  |  |  |  | 0.01522 | 0.00373 | 0.00776 | 0.01651 | 0.00693 | 0.00518 | 0.00711 | 0.00806 | 0.0122 | 0.00212 |
| **Peyrefite** |  |  |  |  |  |  | 0.00286 | 0.003 | 0.03707 | 0.01029 | 0.00601 | 0.01394 | 0.002 | 0.02067 | 0.01065 |
| **Pinnasouk** |  |  |  |  |  |  |  | 0.00374 | 0.03807 | 0.01273 | 0.00596 | 0.00648 | 0.00709 | 0.01159 | 0.00553 |
| **Port de Sète** |  |  |  |  |  |  |  |  | 0.017 | 0.00354 | 0.00384 | 0.00522 | 0.00405 | 0.01303 | 0.00305 |
| **Port-Vendres** |  |  |  |  |  |  |  |  |  | 0.00586 | 0.01097 | 0.01282 | 0.00777 | 0.00319 | 0.01698 |
| **Port Ambonne** |  |  |  |  |  |  |  |  |  |  | 0.00407 | 0.00313 | 0.0022 | 0.00378 | 0.00167 |
| **Port-St-Louis** |  |  |  |  |  |  |  |  |  |  |  | 0.00678 | 0.00319 | 0.00196 | 0.0037 |
| **Thau Eaux blanches** |  |  |  |  |  |  |  |  |  |  |  |  | 0.00514 | 0.00686 | 0.0053 |
| **Thau Lido** |  |  |  |  |  |  |  |  |  |  |  |  |  | 0.00694 | 0.0079 |
| **Thau Mèze** |  |  |  |  |  |  |  |  |  |  |  |  |  |  | 0.0053 |

The * indicates significant values after Bonferroni sequential correction.

**Supplementary Table S3a**: AMOVA analysis of genetic variances within and among localities through the Gulf of Lion. Level of significance is based on 10 000 iterations.

| **Source of variation** | **Sum of squares** | **Variance components** | **Percentage variation** |
| --- | --- | --- | --- |
| **Among localities** | 65.127 | 0.01199 | 0.18 |
| **Among individuals within localities** | 6 305.94 | 0.05517 | 0.84 |
| **Within individuals** | 6 253 | 6.51354 | 98.98 |
| **Total** | 12 624.06 | 6.5807 |  |

**Supplementary Table S3b**: AMOVA analysis of genetic variances within and among localities and type of habitat through the Gulf of Lion. Level of significance is based on 10 000 iterations

| **Source of variation** | **Sum of squares** | **Variance components** | **Percentage variation** |
| --- | --- | --- | --- |
| **Among type of habitat** | 32.273 | 0.00728 | 0.11 |
| **Among localities within type of habitat** | 32.854 | 0.00654 | 0.1 |
| **Among individuals within localities** | 6305.937 | 0.05517 | 0.84 |
| **Within individuals** | 6253 | 6.51354 | 98.95 |
| **Total** | 12 624.06 | 6.58253 |  |


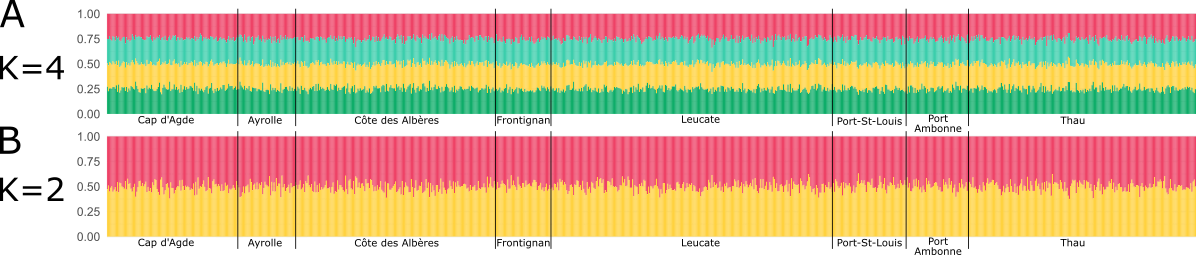


**Supplementary Figure S1**: Cluster analysis obtained from the Bayesian-based method implemented in STRUCTURE v2.3.4 (Pritchard et al., 2000) showing the most likely number of clusters (K) present in P. nobilis populations across the Gulf of Lion resulting from the application of the method described by (A) Evanno et al. (2005) and (B) by Puechmaille (2016).


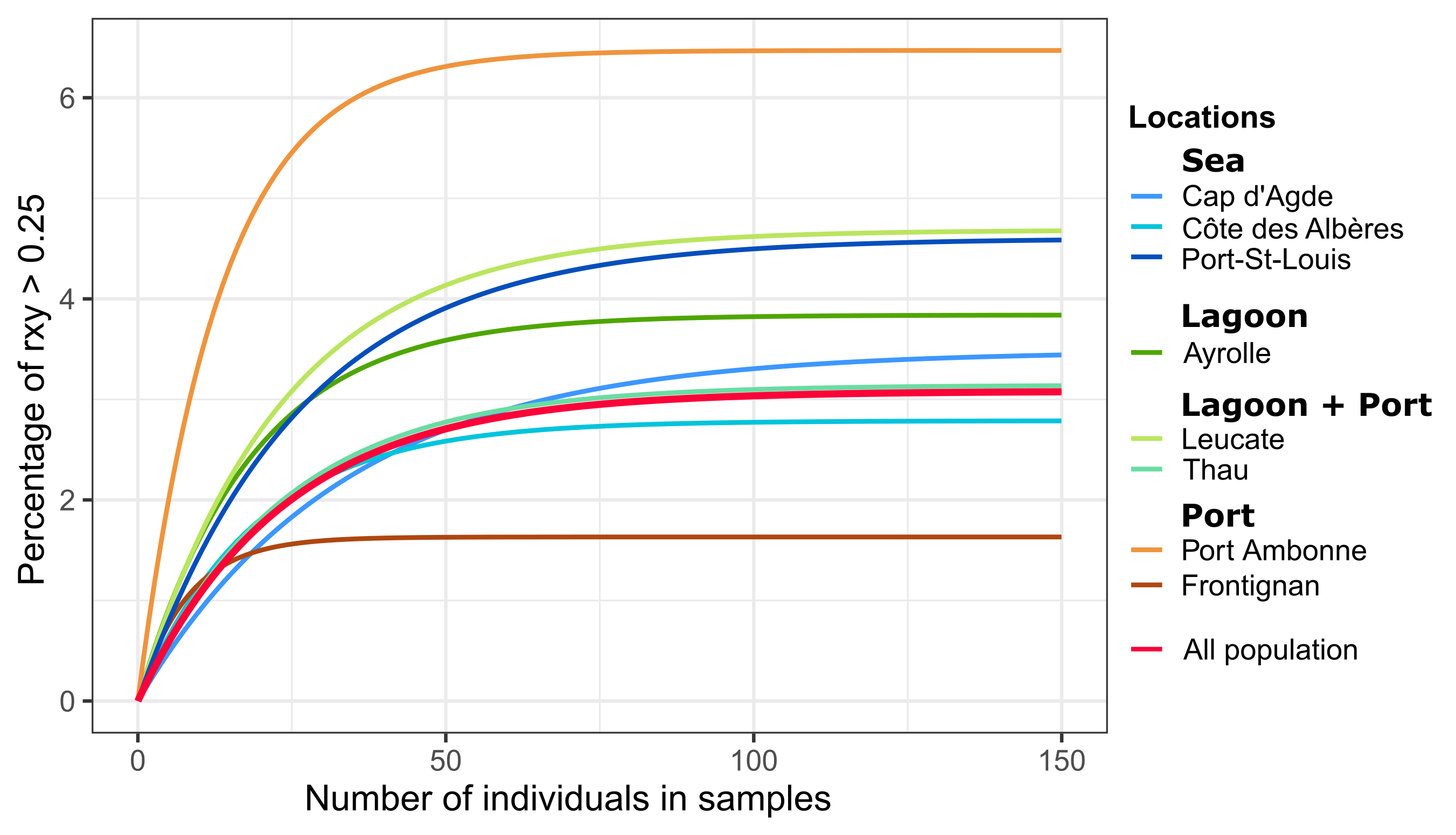


**Supplementary Figure S2**: Relationship between the percentages of related individuals and the number of sampled individuals modeled for each locality.

**Supplementary Table S4**: Percentage of related individuals, sharing at least one common parent (i.e. r_xy_ > 0.25) calculated for each sampled site. Based on Queller and Goodnight (1989) index r_xy_ for relatedness.

| **Locality** | **Site** | **Number of individuals** | **Percentage of related individuals** |
| --- | --- | --- | --- |
| Côte des Albères | Peyrefite | 157 | 3.4 |
|  | Port-Vendres | 19 | 2.3 |
| Leucate | Leucate Chenal | 50 | 4.0 |
|  | Leucate Sud | 53 | 3.9 |
|  | Leucate Port | 105 | 4.5 |
|  | Leucate Nord | 54 | 6.1 |
| Ayrolle | Ayrolle | 51 | 3.5 |
| Cap d'Agde | Cap d'Agde | 116 | 3.4 |
| Port Ambonne | Port Ambonne | 35 | 6.1 |
| Thau | Thau Sud | 47 | 3.3 |
|  | Lido | 54 | 2.5 |
|  | Mèze | 24 | 2.5 |
|  | Eaux Blanches | 34 | 2.0 |
|  | Port de Sète | 61 | 3.1 |
| Frontignan | Frontignan | 49 | 2.1 |
| Port-St-Louis | Port-St-Louis | 51 | 3.6 |
